# SIFR annotator: ontology-based semantic annotation of French biomedical text and clinical notes

**DOI:** 10.1186/s12859-018-2429-2

**Published:** 2018-11-06

**Authors:** Andon Tchechmedjiev, Amine Abdaoui, Vincent Emonet, Stella Zevio, Clement Jonquet

**Affiliations:** 1 Laboratory of Informatics, Robotics and Microelectronics of Montpellier (LIRMM), University of Montpellier, CNRS, 161, rue Ada, 34095 Montpellier cedex 5, France; 2 Center for Biomedical Informatics Research (BMIR), Stanford University, 1265 Welch Rd, Stanford, CA 94305 USA; 3 LGI2P, IMT Mines Ales, Univ Montpellier, Alès, France

## Abstract

**Background:**

Despite a wide adoption of English in science, a significant amount of biomedical data are produced in other languages, such as French. Yet a majority of natural language processing or semantic tools as well as domain terminologies or ontologies are only available in English, and cannot be readily applied to other languages, due to fundamental linguistic differences. However, semantic resources are required to design semantic indexes and transform biomedical (text)data into knowledge for better information mining and retrieval.

**Results:**

We present the SIFR Annotator (http://bioportal.lirmm.fr/annotator), a publicly accessible ontology-based annotation web service to process biomedical text data in French. The service, developed during the *Semantic Indexing of French Biomedical Data Resources (2013–2019)* project is included in the SIFR BioPortal, an open platform to host French biomedical ontologies and terminologies based on the technology developed by the US *National Center for Biomedical Ontology*. The portal facilitates use and fostering of ontologies by offering a set of services –search, mappings, metadata, versioning, visualization, recommendation– including for annotation purposes. We introduce the adaptations and improvements made in applying the technology to French as well as a number of language independent additional features –implemented by means of a proxy architecture– in particular annotation scoring and clinical context detection. We evaluate the performance of the SIFR Annotator on different biomedical data, using available French corpora –Quaero (titles from French MEDLINE abstracts and EMEA drug labels) and CépiDC (ICD-10 coding of death certificates)– and discuss our results with respect to the CLEF eHealth information extraction tasks.

**Conclusions:**

We show the web service performs comparably to other knowledge-based annotation approaches in recognizing entities in biomedical text and reach state-of-the-art levels in clinical context detection (negation, experiencer, temporality). Additionally, the SIFR Annotator is the first openly web accessible tool to annotate and contextualize French biomedical text with ontology concepts leveraging a dictionary currently made of 28 terminologies and ontologies and 333 K concepts. The code is openly available, and we also provide a Docker packaging for easy local deployment to process sensitive (e.g., clinical) data in-house (https://github.com/sifrproject).

## Introduction

Biomedical data integration and semantic interoperability are necessary to enable translational research [1–3]. The biomedical community has turned to ontologies and terminologies to describe their data and turn them into structured and formalized knowledge [4, 5]. Ontologies help to address the data integration problem by playing the role of common denominator. One way of using ontologies is by means of creating semantic annotations. An annotation is a link from an ontology concept to a data element, indicating that the data element (e.g., article, experiment, clinical trial, medical record) refers to the concept [6]. In ontology-based –or semantic– indexing, we use these annotations to “bring together” the data elements from the resources. Ontologies help to design semantic indexes of data that leverage the medical knowledge for better information mining and retrieval. Despite a large adoption of English in science, a significant quantity of biomedical data uses other languages, e.g., French. For instance, clinicians often use the local official administrative language or languages of the countries they operate in to write clinical notes. Besides the existence of various English tools, there are considerably less terminologies and ontologies available in French [7, 8] and there is a strong lack of related tools and services to exploit them. The same is true of languages other than English generally speaking [8]. This lack does not match the huge amount of biomedical data produced in French, especially in the clinical world (e.g., electronic health records).

In the context of the *Semantic Indexing of French Biomedical Data Resources* (SIFR) project (www.lirmm.fr/sifr), we have developed the SIFR BioPortal [9], an open platform to host French biomedical ontologies and terminologies based on the technology developed by the US *National Center for Biomedical Ontology* (NCBO) [10, 11]. The portal facilitates the use and fostering of ontologies by offering a set of services such as search and browsing, mapping hosting and generation, rich semantic metadata description and edition, versioning, visualization, recommendation, community feedback. As of today, the portal contains 28 public ontologies and terminologies (+ two private ones, cf. Table 1), that cover multiple areas of biomedicine, such as the French versions of MeSH, MedDRA, ATC, ICD-10, or WHO-ART but also multilingual ontologies (for which only the French content is parsed) such as Rare Human Disease Ontology, OntoPneumo or Ontology of Nuclear Toxicity.

**Table 1:** SIFR BioPortal semantic resources

Acronym	Name	Source/Group	Format	#Classes/#Individuals	#Props.	Linguality
MDRFRE	Dictionnaire médical pour les activités règlementaires en matière de médicaments	UMLS/UMLS	RRF	68,980	14	FTO
MSHFRE	Medical Subject Headings, version francaise	UMLS/UMLS	RRF	27,879	6	FTO
MTHMSTFRE	Terminologie minimale standardisée en endoscopie digestive	UMLS/UMLS	RRF	1700	1	FTO
STY	Réseau Sémantique UMLS	UMLS/UMLS	OWL	133	0	FTO
CIM-10	Classification Internationale des Maladies - 10ème révision	CISMeF/UMLS	OWL	19,853	0	FTO
WHO-ARTFRE	Terminologie des effets indésirables	CISMeF/UMLS	OWL	3483	0	FTO
CISP-2	Classification Internationale des Soins Primaires, deuxième édition	CISMeF/UMLS	OWL	745	4	FTO
CIF	Classification Internationale du Fonctionnement, du handicap et de la santé	CISMeF/UMLS	OWL	1496	2	FTO
SNMIFRE	Systematized Nomenclature of MEDicine, version française	CISMeF/UMLS	OWL	106,291	8	FTO
MEDLINEPLUS	MedlinePlus Health Topics	CISMeF/UMLS	OWL	849	2	FTO
ATCFRE	Classification ATC (anatomique, thérapeutique et chimique)	CISMeF/UMLS	OWL	5768	2	FTO
PDO	CFEF - Prenatal Diagnosis Ontology	LIMICS	OWL	802	0	FMO
ONTOLURGENCES	Ontologie des urgences	LIMICS	OWL	10,031	61	FMO
CCAM	Classification Commune des Actes Médicaux	CISMeF	OWL	9663	8	FOO
ONTOPNEUMO	Ontologie de la pneumologie française.	LIMICS	OWL	1153	22	FMO
TOP-MENELAS	Top ontologie de ONTOMENELAS	LIMICS	OWL	339	298	FMO
LPP	Liste des Produits et Prestations	AMELI/CISMeF	OWL	3746	4	FOO
NABM	Nomenclature des Actes de Biologie Médicale	AMELI/CISMeF	OWL	1055	3	FOO
INM	Ontologie des Interventions Non Médicamenteuses	CEPS/LIRMM	OWL	159	3	FOO
TRANSTHES	Thésaurus de la transfusion sanguine	INIST-CNRS/Loterre	SKOS	2033	0	FOO
MEMOTHES	Thésaurus Psychologie cognitive de la mémoire humaine	INIST-CNRS/Loterre	SKOS	772	0	FOO
BHN	Biologie Hors Nomenclature	LIRMM/CISMeF	OWL	1534	2	FOO
ONTOTOXNUC	Ontology of nuclear toxicity	CEA/LIMICS	OWL	650	0	FMO
HRDO	Ontologie des maladies rares humaines	INSERM/LIMICS	OWL	135,939	20	FMO
MUEVO	Vocabulaire multi-expertise (patient/médecin) dédié au cancer du sein	LIRMM	SKOS	306	18	FOO
ONL-MR-DA	Ontologie des l’acquisition de jeux de données IRM	NEUROLOG	OWL	702	244	FOO
ONL-DP	Ontologie des traitements de jeux de données	NEUROLOG	OWL	541	220	FOO
ONL-CORE-MSA	Ontologie noyau des instruments pour l’évaluation des états mentaux	NEUROLOG	OWL	329	249	FOO
Average				13,661.2	46.2	
Total				387,623	1206	

One of the main motivation to build the SIFR BioPortal was to design the SIFR Annotator (http://bioportal.lirmm.fr/annotator), a publicly accessible and easily usable ontology-based annotation web service to process biomedical text and clinical notes in French. The annotator service processes raw textual descriptions, tags them with relevant biomedical ontology concepts, expands the annotations using the knowledge embedded in the ontologies and contextualizes the annotations before returning them to the users in several formats such as XML, JSON-LD, RDF or BRAT. We have significantly enhanced the original annotator packaged within the NCBO technology [12, 13], including the addition of scoring, score filtering, lemmatization, and clinical context detection; not to mention some enhancements have not been implemented only for French but have been generalized for the original English NCBO Annotator (or any other annotator based on NCBO technology) through a “proxy” architecture presented by Tchechmedjiev et al. [14]. A preliminary evaluation of the SIFR Annotator has shown that the web service matches the results of previously reported work in French, while being public, of easy access and use, and turned toward semantic web standards [9]. However, the previous evaluation was of limited scope and new French benchmarks have since been published, which has motivated a more exhaustive evaluation of all the new capabilities mostly with the following corpora: (i) the Quaero corpus (from CLEF eHealth 2015 [15]) which includes French MEDLINE citations in (titles & abstracts) and drug labels from the European Medicines Agency, both annotated with UMLS Semantic Groups and Concept Unique Identifiers (CUIs); (ii) the CépiDC corpus (from CLEF eHealth 2017 [16]) which gathers French death certificates annotated with ICD-10 codes produced by the French epidemiological center for medical causes of death (CépiDC^1^). Additionally, the new contextualization features make SIFR Annotator the first general annotation workflow with a complete implementation of the ConText/NegEx algorithm for French [17]; evaluated on two types of clinical text as reported in a dedicated article (Abdaoui et al: French ConText: a Publicly Accessible System for Detecting Negation, Temporality and Experiencer in French Clinical Notes, under review).^2^

The rest of the paper is organized as follows: The Background section presents related work pertaining to ontology repositories, semantic annotation tools, and knowledge-based approaches for French biomedical text information extraction. The Implementation section describes the SIFR BioPortal, the provenance of the ontologies as well as the architecture and implementation details of the SIFR Annotator and its generic extension mechanism. The Results and Evaluation section presents an experimental evaluation of the SIFR Annotator performance through three tasks (named entity recognition, death certificate coding as well as contextual clinical text annotation). The Discussion section analyses the merits and limits of our approach through a detailed error analysis and outlines future directions for the improvement of the SIFR Annotator.

## Background

### Biomedical ontology and terminology libraries

In the biomedical domain, multiple ontology libraries (or repositories) have been developed. The OBO Foundry [18] is a reference community effort to help the biomedical and biological communities build their ontologies with an enforcement of design and reuse principles, which has been a tremendous success. The OBO Foundry web application (http://obofoundry.org) is an ontology library which serves content to other ontology repositories, such as the NCBO BioPortal [10], OntoBee [19], the EBI Ontology Lookup Service [20] and more recently AberOWL [21]. None of these platforms are multilingual or focus on features pertaining to French [22].^3^ Moreover, only BioPortal offers an embedded semantic annotation web service. Another resource for terminologies in biomedicine is the UMLS Metathesaurus [23] which contains six French versions of standard terminologies.

The NCBO BioPortal (http://bioportal.bioontology.org) [10], developed at Stanford, is considered now as the reference open repository for (English) biomedical ontologies that were originally spread out over the web and in different formats. There are 690+ public semantic resources in this collection as of early 2018. By using the portal’s features, users can browse, search, visualize and comment on ontologies both interactively through a web interface, and programmatically via web services. Within BioPortal, ontologies are used to develop an annotation workflow [13] used to index several biomedical text and data resources using the knowledge formalized in ontologies, to provide semantic search features and enhance the information retrieval experience [24]. The NCBO BioPortal functionalities have been progressively extended over the last 12 years, and the platform has adopted semantic web technologies (e.g., ontologies, mappings, metadata, notes, and projects are stored in an RDF^4^ triple store). NCBO technology [11] is domain-independent and open source. A BioPortal virtual appliance^5^ embedding the complete code and deployment environment is available, allowing anyone to set up a local ontology repository and customize it. The NCBO virtual appliance is quite regularly requested by organizations that need to use services like the NCBO Annotator but have to process sensitive data in house e.g., hospitals. NCBO technology has already been adopted for different ontology repositories such as the MMI Ontology Registry and Repository [25], the Earth Sciences Information Partnership earth and environmental semantic portal (see http://commons.esipfed.org/node/1038). We are also working on AgroPortal [26], an ontology repository for agronomy.

As for French, the need to list and integrate biomedical ontologies and terminologies has been identified since the 2000s, more particularly within the Unified Medical Language for French (UMLF) [27] and VUMeF [28] (Vocabulaire Unifié Medical Francophone) initiatives, which aimed to reproduce or get closer to the solutions of the US National Library of Medicine such as the UMLS Metathesaurus [23]. The need to support unified and interrelated terminologies was identified by the InterSTIS project (2007–2010) [29]. This need was to serve the problem of semantic annotation of data. The main results of this project in terms of multi-terminological resources were:

- The SMTS portal based inter alia on ITM technology developed by Mondeca [30]. If SMTS is no longer maintained today, ITM still exists and is deployed by the company for its customers, in the field of health or otherwise.
- The Health Multiple Terminology Portal (HMTP) [31] developed by the CISMeF group, which later became HeTOP (Health Terminology / Ontology Portal – www.hetop.eu) [32]. HeTOP is a multi-terminological and multilingual portal that integrates more than 50 terminologies or ontologies with French content (but only offers public access to 28 of them^6^). HeTOP supports searching for terms, accessing their translations, to identifying the links between ontologies and especially querying the data indexed by CISMeF in platforms such as Doc-CISMeF [33]. The added value of the portal clearly comes from the medical expertise of its developers, who integrate ontologies methodically one by one, produce translations of the terms and index (semi-manually) the data resources of the domain.

The philosophies of HeTOP and NCBO BioPortal are different even if they occupy the same niche. HeTOP’s vision, similar to that of UMLS, is to build a “metathesaurus” so that each source ontology is integrated into a specific (and proprietary) model and is manually inspected and translated. Of course, this tedious work has the added value of a great wealth and confidence in the data integrated, but comes at the cost of a complex and long human process that does not scale to the number of health or biomedical ontologies produced today (similarly, the US National Library of Medicine can hardly keep pace with the production of biomedical ontologies for integration into UMLS). In addition, this content is difficult to export from the proprietary HeTOP information system, which does not offer publicly API or standard and interoperable format for easy retrieval (although, in the context of this work, several ontologies were exported by CISMeF in OWL format thanks to a wrapper developed during the SIFR project). The vision of the NCBO BioPortal is different, it consists in offering an open platform, based on semantic web standards, but without integrating ontologies one by one in a meta model. The platform supports mechanisms for producing and storing alignments and annotations but does not create new content nor curate the content produced by others. The portal is not multilingual, but it offers a variety of services to users who want to upload their ontologies themselves or just reuse some already stored in the platform. For an exhaustive comparison of HeTOP and BioPortal annotation tools, we recommend reading [34].

Within the SIFR project, we were driven by a roadmap to (i) make BioPortal more multilingual [22] and (ii) design French-tailored ontology-based services, including the SIFR Annotator. We have reused NCBO technology to build the SIFR BioPortal (http://bioportal.lirmm.fr) [9], an open platform to host French biomedical ontologies and terminologies only developed in French or translated from English resources and that are not well served in the English-focused NCBO BioPortal. The SIFR BioPortal currently hosts 28 French-language ontologies (+ two privates) and comes to complement the French ecosystem by offering an open, generic and semantic web compliant biomedical ontology and health terminology repository.

### Annotation tools for French biomedical data

One of the main use cases for ontology repositories is to allow the annotation of text data with ontologies [6], so as to make the formal meaning of words or phrases explicit (structured knowledge) through the formal structure of ontologies, which has numerous applications. One such application is semantic indexing, where text is indexed on the basis of annotated ontology concepts, in such a way as to allow information retrieval and access through high level abstract queries, or to allow for semantically enabled searching of large quantities of text [35]. For example, when querying data elements, one may want to filter search results by selecting only elements that pertain to “disorders” by performing a selection through the relevant semantic annotations with UMLS Semantic Group [36] or Semantic Types [37]. In this article, we mainly focus on annotation tools for French biomedical data.^7^

Ontology-based annotation services often accompany ontology repositories. For instance, BioPortal has the NCBO Annotator [12, 13], OLS had Whatizit [38] and now moved to ZOOMA, and UMLS has MetaMap [39]. Similarly, since 2004, the CISMeF group has developed several French automatic indexing tools based on a bag of words algorithm and a French stemmer. We can mention: (i) F-MTI (French Multi-Terminology Indexer) now property of Vidal, a French medical technology provider [40]. (ii) the ECMT (Extracteur de Concepts Multi-Terminologique – http://ecmt.chu-rouen.fr) web service, the core technology of which has been transferred to the Alicante company. As a quick comparison, ECMT does not allow to choose the ontology to use in the annotation process, offers only seven terminologies, and supports semantic expansion features (mappings, ancestors, descendants) only since v3 (released after the start of SIFR project). The web service does not follow semantic web principles, does not enforce the use of URIs and the public fronting API is limited to short snippets of text. However, both F-MTI and ECMT’s use of a more advanced concept matching algorithm based on natural language processing techniques (bag of words) is an advantage compared to the SIFR Annotator.

A quantitative evaluation of annotation performance is of critical importance to enable comparison to other state-of-the-art annotation systems. In the following, we shall review existing evaluation campaigns for French biomedical Named Entity Recognition (NER)^8^ and a brief qualitative and quantitative comparison of participating systems.

Since 2015, the main venue for the evaluation of French biomedical annotation are the CLEF eHealth information extractions tasks [16, 41, 42]. In 2015 (Task1b) and 2016 (Task2), the objective was to perform biomedical entity recognition on the French-language Quaero corpus [15], which contains two sub-corpora: *EMEA* (European Medicines Agency), composed of 12 training drug notices and four test notices; and *MEDLINE* composed of 832 citation titles for training and of 832 titles for testing. The objective of the task was twofold: 1) to annotate the input text with concept spans and UMLS Semantic Groups (called *plain entity recognition or PER*); 2) annotate previously identified entities with UMLs CUIs (called *normalized entity recognition or NER*). The 2016 edition repeated the same task with a different subset of training documents (the training corpus of 2016 was the test corpus of 2015) and test sets. In 2016, there was also a second annotation task, where the aim was to annotate each line of a French death certificates corpus with ICD-10 diagnostic codes (the test corpus contains 31 k certificates and 91 k lines). The 2017 edition (task 2) kept only the death certificate annotation task, although corpora were proposed in both French and English.

The participating systems included a mixture of machine learning methods and knowledge-based annotation methods. In 2015, there were two knowledge-based systems, ERASMUS [43] and SIBM (CISMeF) [44]. The ERASMUS system ranked first with a F1 score of over 75%; it used machine translation (concordance across two translation systems) to translate UMLS concept labels and definitions into French before applying an existing English biomedical concept recognition tool with supervised post-processing. The CISMeF system was based on their ECMT annotation web service using a dictionary composed of concept labels from French biomedical ontologies from HeTOP (55 of them at that time, extended from the seven accessible in the public ECMT web service), and obtains variable evaluation results ranging from under 1% F1 score to 22% depending on the task and parameters of the evaluation (up to 65% approximate match F1-score). The other participating systems were mostly based on *conditional random fields* or classifier ensemble systems and ranked competitively with the ERASMUS system.

In 2016, ERASMUS and SIBM (CISMeF) participated again [45, 46]. SIBM (CISMeF) participated with an entirely different knowledge-based annotation system. Both SIBM and ERASMUS, along with BITEM, performed concept matching from the French subset of UMLS. The other participating systems were based on supervised machine learning techniques (*support vector machines, linear dirichlet allocation, conditional random fields*) but only participated for plain entity recognition. The ERASMUS system prevailed once more using the same approach as in 2015 with F1 scores comprised between 65 and 70% on PER and 47% and 52% for NER. The SIBM system from CISMeF performed much better than in 2015 with F1 scores between 42 and 52% for PER and between 27 and 38% for NER depending on the task (up to 66% approximate match F1 score).

For both 2015 and 2016, knowledge-based systems tend to perform better than supervised systems, in particular ERASMUS’s machine translation approach. Supervised systems are only competitive against plain entity recognition, they are otherwise outclassed, likely due to the relatively small amount of training data available. Systems relying only on French terminologies (mostly every system except ERASMUS) tend to be at a disadvantage, as the coverage of corpus by French labels is low, given that the corpus was built by bilingual annotators that did not restrict themselves to French labels and used CUIs to annotate sentences independently of the existence of a label in French for those CUIs in UMLS. This limitation also concerns the SIFR Annotator which uses only French terminologies; we will discuss later how we address this bias in our evaluation.

In 2016, for the death certificate annotation task, the ERASMUS system prevailed, but this time using an information retrieval indexing approach (Solr indexing + search on lines) with over 84% F1 score. Follow, ERIC-ECSTRA (a supervised system) [47], SIBM, LIMSI (information retrieval approach, [48]) and BITEM (pattern matching between dictionary and text).

In 2017, there were a total of seven systems, including our generic SIFR Annotator; comparison results are reported in the Results section of this article. Among the seven systems, six were knowledge-based. LITL [49] used a Solr index to create a term index from the provided dictionaries and a rule-based matching criterion based on index searches. We (LIRMM) [50] used the SIFR Annotator with an additional custom terminology generated from the provided dictionaries. Mondeca [51] also used the dictionaries along with a GATE annotation workflow [52] to match codes to sentences. SIBM [53], dropping the ECMT-based system, matched terms with multiple level (word, phrase) fuzzy matching and an unsupervised candidate ranking approach (for disambiguation), similarly to WBI [54] that used a Solr index and fuzzy search to match candidates along followed by supervised candidate ranking.

Most of CLEF eHealth’s French information extraction approaches were specific to the evaluation tasks. While they are interesting to push the state-of-the-art and obtain the best performance within a competitive context, their general usefulness outside of the task is limited. The custom systems implemented to best fit the tasks are not easily generalizable for use outside of the competition as independent, open and generic systems. In 2015 and 2016, only SIBM used a generic approach not specific to the benchmark. In 2017, SIBM switched to a task-specific approach and SIFR Annotator was the only open and generic approach, and which is available as an open web service independently of the competition. In this article, we report on how we exploited the task as a means of evaluating and mitigating the shortcoming of the SIFR Annotator in order to implement or identify improvements to the annotation service generalizable to any application of biomedical semantic annotation.

The CLEF eHealth 2017 Task 1 also included a reproducibility track, where participants could submit instructions to build and run their systems and evaluate the reproducibility of each other’s experiments. Four participating systems partook in this exercise (KFU, LIRMM, the unofficial LIMSI and UNIPD, another non-official participant). The evaluation consisted of allocating a maximum of 8 h per system to replicate the results and to fill in an evaluation survey by reporting difficulties and observations. Our SIFR Annotator system produced results with under 1% difference in precision, recall of F1 sore compared to our official submission. While our CLEF eHealth experiments were performed in a sandboxed and controlled environment (clean instance of SIFR Annotator with only the terminologies needed for the evaluation), we decided to instruct reproducing teams how to use our online production SIFR Annotator for the reproduction to demonstrate the robustness of the platform and its ease of access/usability. The reproduction was successful and led to an accurate reproduction of the sandboxed results within less than an hour for reproducing teams.

## Implementation

### Building the SIFR BioPortal

#### Terminology/ontology acquisition

Porting an ontology-based annotation tool to another language in only half of the work. Beyond specific matching algorithms, one of the main requirements is to gather and prepare the relevant ontologies and terminologies used in the annotation process. Indeed, the ontologies offer thematic coverage, lexical richness and relevant semantics. However, ontologies and terminologies in biomedicine are spread out over the Web, or not yet publicly available; they are represented in different formats, change often and frequently overlap. In building the SIFR BioPortal and Annotator our vision was to embrace semantic web standards and promote openness and easy access. The list of ontologies and terminologies currently available in the SIFR BioPortal is available in Table 1. Hereafter, we describe each of the sources:

- Our first source of semantic resources is the UMLS Metathesaurus, which contains six French terminologies, translations of their English counterparts. For instance, the MeSH thesaurus is translated and maintained in French by INSERM (http://mesh.inserm.fr) and new releases are systematically integrated within the UMLS Metathesaurus. We used the NCBO-developed umls2rdf tool (https://github.com/ncbo/umls2rdf) to extract three of these sources in RDF format and load them in our portal.^9^ These sources are regularly updated when they change in the UMLS.
- Our second source of French terminologies is the CISMeF group, which in France is the most important actor to import and translate medical terminologies. During the SIFR project, the group developed an OWL extractor for the HeTOP platform which can be used to produce an OWL version of any resource integrated by CISMeF within HeTOP. 11 of the SIFR BioPortal terminologies have been produced with this converter and rely on CISMeF for updates, URI providing and dereferencing.
- Our third source of ontologies is the NCBO BioPortal. Indeed, multilingual biomedical ontologies that contain French labels are generally uploaded to the NCBO BioPortal by their developers. We automatically pulled the ontology sources into the SIFR BioPortal and display/parse only the French content in our user interface and backend services (including the SIFR Annotator dictionary). By doing so, the NCBO BioPortal remains the main entry point for such ontologies –for English use cases– while SIFR BioPortal serves the French content of the same ontologies and links back to the mother repository. Ontology developers do not have to bother about the SIFR BioPortal as the source of information for ontology metadata and new versions remains the NCBO BioPortal.
- Finally, direct users or institutions are the last source of ontologies and terminologies in the SIFR BioPortal. The resources concerned are semantic resources developed only in French that are either not included in HeTOP or not offered by CISMeF. Indeed, such use-cases are outside the score of CISMeF with their HeTOP plaform and adding new ontologies to HeTOP involves a lengthy administrative process. Therefore, the SIFR BioPortal fills this need for the French biomedical ecosystem by offering an open and generic platform on which uploading a resource is quick and obvious and automatically comes to complete the SIFR Annotator dictionary. For instance, the CNRS’s Scientific and Technical Information Department helps scientists in adopting semantic web standards for their standardized terminologies used for instance in literature indexing. The Loterre project (www.loterre.fr) offers multiple health related SKOS vocabularies for which the SIFR BioPortal is another point of dissemination and automatic API access.

#### Portal content and ontology curation

Within the SIFR BioPortal, semantic resources are organized in groups. Groups associate ontologies from the same project or organization for better identification of their provenance. For instance, we have created a group for all the ontologies of the LIMICS research group, imported from the NCBO BioPortal, or being a translation of an English UMLS source. The SIFR BioPortal has the capability (inherited from the NCBO BioPortal) to classify concepts based on CUIs and Semantic Types from UMLS. For instance, it enables the SIFR Annotator to filter out results based on a certain Semantic Types of Semantic Groups (as described later). For the three terminologies within the UMLS group directly extracted from the UMLS Metathesaurus format (MDREFRE, MSHFRE, MTHMSTFRE) the CUI and Semantic Type information provided by the Metathesaurus were correctly available. However, for most of the six other ontologies in the UMLS group, produced by CISMeF in OWL format (CIM-10, SNMIFRE, WHOART-FRE, MEDLINEPLUS, CISP-2, CIF), the relevant UMLS identifiers (CUI & TUI) were missing or improperly attached to the concepts. We therefore enriched them to reconcile their content with UMLS concepts and Semantic Type identifiers [55]. For this, we used a set of previously reconciled multilingual mappings [56] made through a combination of matching techniques to associate concept codes between French terminologies and their English counterparts in UMLS.

All in all, the SIFR BioPortal contains now 10 ontologies with UMLS interoperability among a total of 28. Since we relied on retrieving and normalizing existing mappings, we could only enrich ontologies that were in UMLS to begin with, however, we are working on integrating a generalized reconciliation feature that would automatically align terminologies submitted to SIFR BioPortal with the UMLS Metathesaurus. In addition, SIFR BioPortal includes an interlingual mapping feature that allows interlinking with equivalent ontologies in English. There are currently nine French terminologies with interportal mappings to NCBO BioPortal [56]. In a broader multilingual setting, the UMLS Metathesaurus, for some resources such as MeSH, is a de-facto multilingual pivot that allows linking annotations with concepts across languages and to generate inter-portal mappings. As with any multilingual pivot structure, care must be taken when dealing with ambiguous multilingual labels that may be an important source of noise if more than two languages are involved.

There are numerous practical and tedious technical issues with any efforts to integrate biomedical ontologies in an open ontology repository. Heterogeneous ontologies often contain many inconsistencies and “incorrect” constructs which often show up when put together in the same platform. For instance:

- Inconsistent concept hierarchy (multiple roots, no hierarchy, no root concept);
- Non-compliance with best practice standards (especially semantic web standards);
- Use of heterogeneous and non-standard properties.

Moreover, ontologies, although they may be available online, often do not define clear licensing information, which prevents their diffusion on any ontology library. Lengthy investigations to find the authors (or authority organization) of the ontologies and then to negotiate licensing terms are often required before a resource can be hosted in the SIFR BioPortal. In certain cases, the semantic resource is accessible (user interface & web services) but not downloadable.

Despite the numerous challenges facing such an endeavor, SIFR BioPortal, across all the ontologies indexed in the repository, currently represents the largest open French-language biomedical dictionary/term repository,^10^ with over 380 K concepts and around twice that number of terms. Enabling the SIFR Annotator service to use additional ontologies is as simple as uploading them to the portal (the indexing and dictionary generation are automatic) and take only a few minutes. Table 1 summarizes some statistics about the repository’s content in terms of size and general characteristics of the semantic resources.

On the subject of licencing of the resources, two of the four terminologies directly extracted from UMLS are subjected to UMLS license terms and are not directly downloadable from SIFR BioPortal. They are available for people that do have UMLS licenses, although our system doesn’t directly interface with the UMLS license server.

For the other ontologies and terminologies, access rights have been discussed to allow us to make them openly available when relevant. Often, resources within SIFR are loaded by their developer directly. We encourage our contributors to unambiguously assign a specific license to their ontology or terminology (and provide the technical means to capture this information). In addition, there are some private ontologies that are not visible to the public, any user can add such ontologies for their private needs and access is granted only by the user who submitted the ontology.

It is important to note that regardless of licensing, the non-private resources can always be used for annotation i.e., their identifiers (URI, CUI) can be used to annotate text sent to the Annotator.

### SIFR Annotator Workflow & Features

**Fig. 1:**
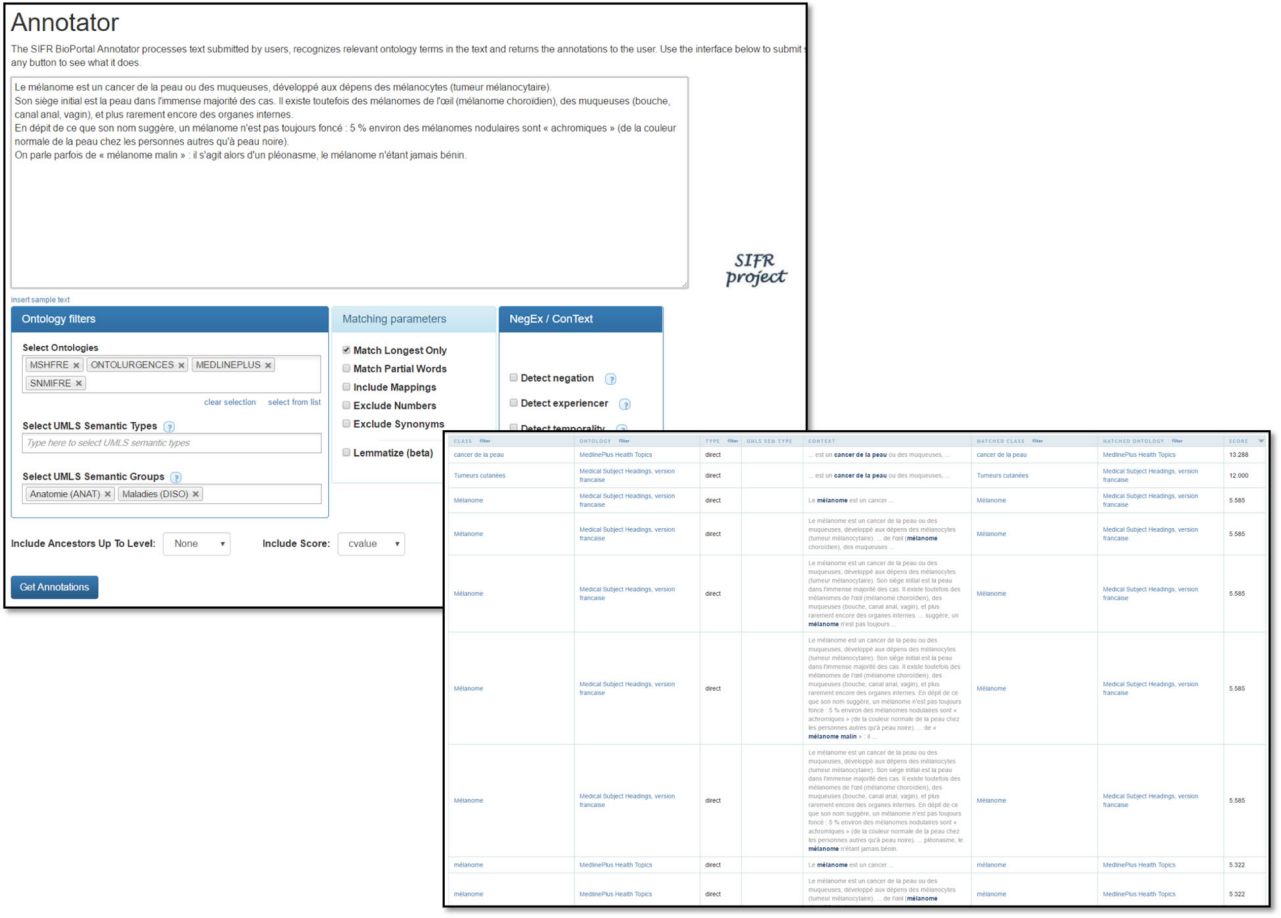
The SIFR Annotator user interface. The upper screen capture illustrates the main form of the annotator, where one inputs text and selects the annotation parameters. The lower screen capture shows the table with the resulting annotations

The SIFR Annotator allows annotating text supplied by users with ontology concepts. It uses a dictionary composed of a flat list of terms built from the concept and synonym labels from all the ontologies and terminologies uploaded in the SIFR BioPortal. The SIFR Annotator is built on the basis of the NCBO Annotator [12, 13] which is included in the NCBO virtual appliance. We have customized the original service for French but also developed new language independent features. In the following, we describe the complete SIFR Annotator workflow (including new and preexisting functionalities). The Annotator is meant to be accessed through a REST API but there is also a user interface that serves as a demonstrator and that allows a full parametrization (Fig. 1).

The SIFR Annotator mainly relies on Mgrep [57] as concept recognizer. Although experiments have been carried out –both by NCBO and us– to swap the underlying concept recognizer with another (MetaMap, Alvis, Mallet, UniTex), Mgrep is still the default recognizer. It uses a simple label matching approach but offers a fast and reliable (precision) matching that enables its use in real-time high load web services. Mgrep and/or the NCBO Annotator have been evaluated [58–61] on different English-language datasets and usually perform very well in terms of precision e.g., 95% in recognizing disease names [62]. A comparative evaluation of MetaMap [39] and Mgrep within NCBO Annotator was made in 2009 [12] when the NCBO Annotator was first released. There are, however, no evaluations of Mgrep on French text.

**Fig. 2:**
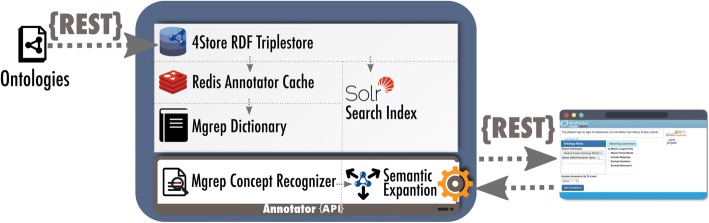
NCBO and SIFR Annotator(s) core components

The architecture of the NCBO and SIFR Annotator(s) is described in Fig. 2. When ontologies are submitted to the corresponding repository, they are loaded in a 4Store RDF triplestore and indexed in an Apache Solr search index. Subsequently, the labels of concepts (main labels and alternative labels) are cached within a Redis table, and thereafter used to generate a dictionary for the Mgrep concept recognizer. During annotation, the concepts that have been matched to the text undergo semantic expansion (mappings and hierarchy). The process and associated features are detailed hereafter with a running example to illustrate the steps more concretely.

#### Dictionary creation

The dictionary consisting of all the terms harvested from the ontologies is a central component of the concept recognizer. Mgrep works with a tab-separated dictionary file containing unique identifiers for each term as well as the term to match themselves. If terms are duplicated among multiple ontologies, they will be repeated inside the Mgrep dictionary.

When a new ontology is uploaded and parsed by the SIFR BioPortal concept labels and synonyms are indexed (using Solr) and cached (using Redis) for respectively faster retrieval and to build the dictionary. For features such as lemmatization another custom lemmatized dictionary is also produced and used depending on the annotation options selected.

For instance, the MSHFRE concept D001943^11^ with preferred label “Tumeurs du sein” and three synonyms will correspond to the following entries in the default dictionary:

- 18774661661 tumeur du sein
- 18774661661 carcinome mammaire humain
- 18774661661 cancer du sein
- 18774661661 tumeurs mammaires humaines

In this example, the entries in the lemmatized dictionary would be singular.

To augment our Annotator's recall performance, we have implemented some heuristics to extend the dictionary:

- Remove “SAI”/“Sans précisions”/“Sans autre précisions”/“Sans explications”/“Non classés ailleurs”at the end of the concept labels as they are superfluous for annotation. For example, “insuffisance hépatique, sans précision” becomes “insuffisance hépatique”.
- Strip diacritics from accented characters, e.g., “insuffisance hépatique” becomes “insuffisance hepatique”.
- Separate individual clauses from conjunctive sentences (split on by coordinating conjunctions), e.g., “absence congénitale de la vessie et de l’urètre” becomes “absence congénitale de la vessie” and “absence congénitale de l’urètre”.
- Normalize punctuation (replace by spaces).
- Remove parenthesized or bracketed precisions, e.g., “myopathie myotubulaire (centro-nucléaire)” becomes “myopathie myotubulaire”.

Our experiments have shown that recall increases with such heuristics, while precision decreases. Given that splitting labels increases noise, the heuristics are currently deactivated by default. For example, the dictionary entry:

- 77366455283 **Troubles généraux et anomalies au site d'administration**

Would be split as follows after the application of the heuristics:

- 77366455283 **Troubles généraux au site d'administration**
- 77366455283 **anomalies au site d'administration**

Possibly generating false positive annotations.

**Fig. 3:**
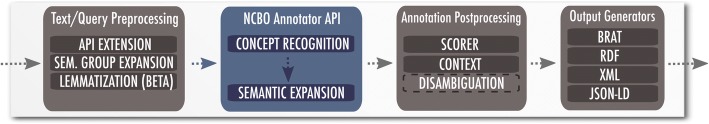
Proxy service architecture implementing the SIFR Annotator extended workflow. During preprocessing, parameters are handled and text can be lemmatized, before both are sent to the core annotator components. During annotation postprocessing, scoring and context detection are performed. Subsequently, the output is serialized to the requested format

The NCBO Annotator is developed and maintained by the NCBO and does not easily support quick add-ons. To extend the original Annotator’s architecture without modifying the original application, we developed a proxy web service that can run independently and extend the service by pre-processing inputs and post-processing outputs, as we will discuss further in Section “Generalization to the any NCBO-like Annotator”. Figure 3 describes the extended SIFR Annotator workflow, where the blue frame represents the core components from Fig. 2. The main steps of the workflow are described in more detail hereafter.

#### Text/query preprocessing

When a query is sent to the SIFR Annotator, it first performs some preprocessing on the parameters to implement some of the extended features e.g., lemmatizing the text. At this stage, some parameters are intercepted and others are rewritten to be forwarded. For example, Semantic Groups are expanded into appropriate Semantic Types that are then handled by the original core Annotator components. For instance, to annotate the text “diagnostic de cancer du sein précoce” with MeSH and Meddra and with concepts belonging to the ‘disorders’ Semantic Group, one will make the following request to SIFR Annotator:

- text = “diagnostic de cancer du sein précoce”
- ontologies = “MSHFRE,MDRFRE”
- semantic_groups = DISO.^12^

During this step, the latest parameter will be transformed into a list of Semantic Types (**T020,T190,T049,T019,T047,T050,T033,T037,T048,T191,T046,T184**) for “disorder” that are handled by the original annotator web service (described hereafter).

#### Core annotator components

At this step the original core components inherited from the NCBO technology are called:

- Concept recognition. The text is first passed to the concept recognizer, by default Mgrep, along with the previously generated dictionary. Mgrep, returns an annotation with the following information: concept identifier and the substring of the text corresponding to the matched token with its start-end offsets (from the beginning of the text in number of characters). The Annotator then retrieves the information (particularly URIs) of each annotating concept in the Solr index in order to generate a significant response to the users. Concept recognition can be parameterized with:
  - ○ match_longest_only = true*.* Keeps the longest annotation spans, among overlapping annotations. For example, if we annotate “cancer du sein”, this parameter will discard the individual “sein” and “cancer” annotations.
  - ○ match_partial_words = true*.* Enables matching concepts that correspond to substrings in tokens. For example, for the text “système cardiovasculaire”, we would match the concept “vasculaire” when this option is enabled.
  Other secondary parameters are available (e.g., stop words, minimum token length, inclusion/exclusion of synonyms).^13^
- Annotation filtering. The SIFR Annotator can filter annotations by UMLS Semantic Types and UMLS Semantic Groups for resources with concepts enriched with such information; typically, those from the UMLS group.
  - ○ semantic_types = [list_of_TUIs], semantic_groups = [list_of_SemGroups]^14^
  For instance, a pharmacogenomics researcher doing a study, may restrict the annotations to the types ‘disorders’ and ‘chemicals & drugs’ to investigate the effect of adverse drug reactions.
- Semantic expansion. Direct annotations identified within the text are then expanded using the hierarchical structure of ontologies as well as mappings between them. For example: an is-a transitive closure component traverses an ontology parent-child hierarchy to create new annotations with parent concepts. For instance, if a text is annotated with a concept from HRDO, such as mélanome, this component generates a new annotation with the term Tumeur/néoplastie, because HRDO provides the knowledge that a melanoma is a kind of neoplasm/tumor. Similarly, the mapping component will create additional annotations with ontology concepts mapped to the previously matched annotating concepts. This functionality allows to “expand” the lexical coverage of an ontology by using alignments with more lexically rich ontologies. Or it enables the SIFR Annotator to use the semantics of other ontologies while returning annotations with solely the user selected target ontologies. For instance:
?text=Néoplasme malin_&longest_only=true
&expand_mappings=true
&expand_class_hierarchy=true
&class_hierarchy_max_level=1

In this example, “Néoplasme malin” directly matches only in SNMIFRE, however the SNMIFRE concept maps to 7 other ontologies through mappings (CUI mappings from UMLS and user-contributed mappings). This means that if we need to use, for instance, MeSH (MSHFRE) as an annotation target, the mappings will enable us to perform concept recognition with the full richness of the labels of equivalent concepts through said mappings, while returning only annotations with MeSH concepts to the user.

The UMLS Metathesaurus, for some resources such as MeSH is a de-facto multilingual pivot that allows expanding annotations with concepts across languages. As with any multilingual pivot structure, care must be taken when dealing with ambiguous multilingual labels that may be an important source of noise.

#### Annotation Postprocessing

Annotations resulting from concept recognition and semantic expansion are post-processed –expanded, filter or enriched. Clinical context detection and scoring are two examples of annotation enrichment, while score-threshold and Semantic Group filtering are examples of filtering operations.

- Scoring. When doing ontology-based indexing, the scoring and ranking of the results become crucial to distinguish the most relevant annotations within the input text. For instance, one may assume a term repeated several times will be of higher importance. Higher scores reflect more important or relevant annotations. However, this feature is not included in the NCBO Annotator.^15^ In the SIFR Annotator, we have implemented and evaluated a new scoring method allowing to rank the annotations and enabling to use such scores for better indexing of the annotated data. By using a natural language processing-based term extraction measure, called C-Value [63], we were able to offer three relevant scoring algorithms which use frequencies of the matches and positively discriminate multi-words term annotations. This work is reported and evaluated in Melzi et al. [63]. We also implemented a thresholding feature that allows to prune annotations based on absolute or relative score values^16^:
  - ○ score = [cvalue, cvalueh, old] allows to select the scoring method.
  - ○ score_threshold = [0–9] + sets an absolute score cut-off threshold. Annotations with lower scores are discarded.
  - ○ confidence_threshold = [0..100] sets a relative cut-off threshold on the score density function for the distribution of annotation scores returned by the annotator.
- Clinical context detection. When annotating clinical text, the context of the annotated clinical conditions is crucial: Distinguishing between affirmed and negated conditions (e.g., “no sign of cancer”); whether a condition pertains to the patient or to others (e.g., family members); or temporality (is a condition recent or historical or hypothetical). NegEx/ConText, is one of the best performing and fastest (open-source) algorithms for clinical context detection in English medical text [64, 65]. NegEx/ConText is based on lexical cues (trigger terms) that modify the default status of medical conditions appearing in their scope. For instance, by default the system considers a condition affirmed, and marks it as negated only if it appears under the scope of a negation trigger term. Each trigger term has a pre-defined scope either forward (e.g., “denies”) or backward (e.g., “is ruled out”), which ends by a colon or a termination term (e.g., “but”). Although an implementation of NegEx was available for French [66], we extended it to the complete ConText algorithm by methodologically translating and expanding the required trigger terms. We integrated NegEx/ConText in SIFR Annotator, which is now a unique open ontology-based annotation service that both recognize ontology concepts and contextualize them. This work is reported and evaluated in detail in Abdaoui-et-al.; however, we briefly report performance evaluation in Section “Clinical Context Detection Evaluation”. Here is an example where all three context features are enabled:
  - ?text=Le patient n'a pas le cancer, mais son père a des antécédents de mélanome
  - &negation=true
  - &experiencer=true
  - &temporality=true
  - &semantic_groups=DISO

#### Output generators

Finally, the workflow generates the final JSON-LD output or converts it to different formats (e.g., BRAT). NCBO Annotator supports JSON-LD and XML outputs, but while JSON-LD is a recognized format, it is not sufficient for many annotation benchmarks and tasks, especially in the semantic web and natural language communities. SIFR Annotator adds support for standard linguistic annotation formats for annotation (BRAT and RDF) and task-specific output formats (e.g., CLEF eHealth/Quaero). The new output formats allow us to produce outputs compatible with evaluation campaigns and in turn to evaluate the SIFR Annotator. Moreover, they enable interoperability with various existing annotation standards.

For instance, in order to generate the output for the Quaero evaluation, one may use:

- ?text=cancer_du_poumon
- &semantic_groups=DISO
- &format=quaero

### Generalization to the any NCBO-like annotator

In order to generalize the features developed for French in the SIFR BioPortal to annotators in other BioPortal appliences, we have adopted a *proxy*^17^ architecture (presented previously), that allows the implementation of features on top of the original REST API, thereby extending it through an intermediary web-service. The advantage of such an architecture is that a proxy instance can be seamlessly pointed to any running BioPortal instance. We have set-up this technology to port new features to the original BioPortal service and offer an NCBO Annotator+ [14] and to the AgroPortal [26]. Hereafter is an example of an annotation request on an English sentence sent to the NCBO Annotator+ using the extended features enabled by the proxy architecture:

- http://services.bioportal.lirmm.fr/ncbo_annotatorplus/
- ?text=The patient has no sign of melanoma but his father had history of skin cancer.
- &ontologies=MESH
- &longest_only=true
- &negation=true
- &experiencer=true
- &temporality=true
- &score=cvalue
- &semantic_groups=DISO

## Results and evaluation

In this section we shall present and analyze our evaluation of SIFR Annotator on three tasks. The first is biomedical named entity recognition and normalization (using the Quaero corpus from CLEF eHealth 2015), the second is ICD-10 diagnostic coding of death certificates (using the CépiDC corpus from CLEF eHealth 2017) and the third is a summary of the evaluation for the context detection features of SIFR Annotator (negation, temporality, experiencer). We evaluate each feature independently: the purpose of the two first evaluations is to gauge how the SIFR Annotator performs for concept recognition; while the third evaluation assess the accuracy of our French adaptation of ConText.

### Annotation of MEDLINE titles and EMEA notices with UMLS concepts and semantic groups

As discussed in Section “Annotation Tools for French Biomedical Data”, the only French biomedical named entity recognition openly available corpora come from the CLEF eHealth information extraction tasks. The CLEF eHealth NER tasks from 2015 and 2016 tasks are based on subsets of the Quaero corpus [15]. We evaluate the ability of SIFR Annotator to identify entities and annotate them with UMLS Semantic Groups (Plain Entity Recognition or PER evaluation) and CUIs (Normalized Entity Recognition or NER evaluation) on the subset of the Quaero corpus comparable to the results of CLEF eHealth 2015 Task 1 (training corpus in Quaero).

**Fig. 4:**
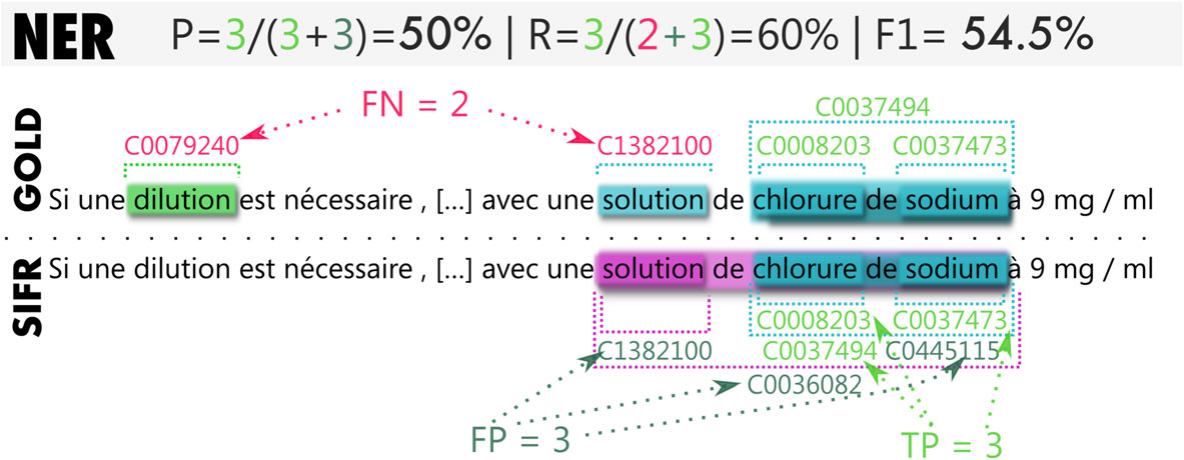
Illustration of the PER annotation task and the score computation. Entities in PER are identified by their character offsets (begin and end from the start of the text) and by their UMLS Semantic Group

**Fig. 5:**
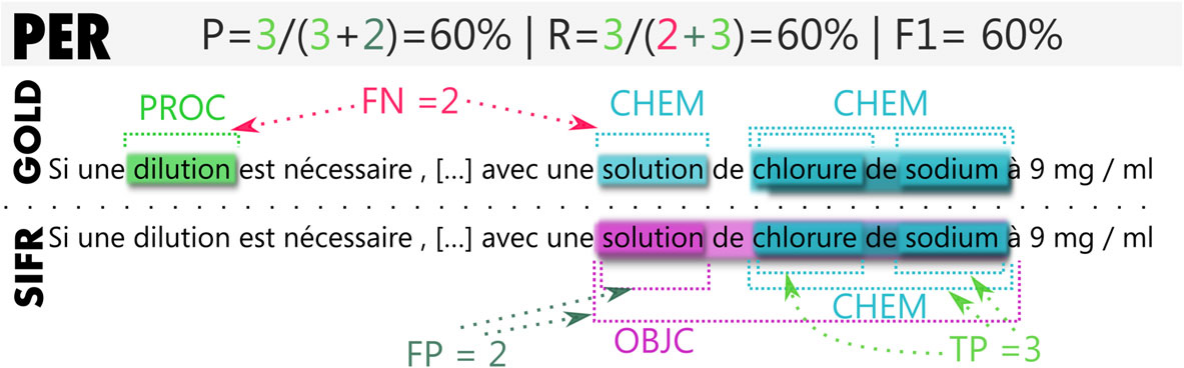
Illustration of the NER annotation task and the score computation. In NER, we annotate entities found in PER with one or more CUIs

Figure 4 illustrates the objective of the PER evaluation task and Fig. 5 that of the NER evaluation tasks (and their score calculation). The example is an actual sample from the results produced by SIFR Annotator and illustrates some of the limitations of the evaluation. In Plain Entity Recognition, some entities are not contained in the semantic resources of the SIFR BioPortal (dilution), some entities are recognized properly, but are categorized in a different Semantic Group due to ambiguity (for “solution”, both classifications (CHEM, OBJC) are often correct but the gold standard keeps only one), some entities are recognized by SIFR Annotator but are not contained in the gold standard (although they could or should like,“solution de chlorure de sodium” in the example, which is the longest possible match).

For the normalized entity annotation with CUIs, if the entity and its Semantic Group are wrong, a false positive is generated, even if the CUI is actually correct (e.g., “solution” C1282100). Which is likely to lead to overall reductions in precision compared with the PER evaluation.

Additionally, the SIFR Annotator may identify several valid CUIs, although the gold standard always expects a single one (non-exhaustive annotation). For example, the software annotates “chlorure de sodium” with C0037494 and C0445115. The former is what the gold standard expects, the CUI for the chemical solution, while the latter is the CUI for the pharmaceutical preparation (normal saline), which is a correct answer that counts as a false positive.

#### Construction Biases & Production of the adapted Quaero Corpus

As previously mentioned, one important bias of Quaero, is that it uses UMLS meta-concepts identified by CUIs irrespective of whether or not a French label exists in the UMLS. We have seen that this had a strong influence on the results and constitutes and advantage for systems using machine translation (ERASMUS in particular).

By reconciling UMLS concepts and Semantic Type information inside the French terminologies offered by CISMeF [55], we have mitigated this issue by greatly extending the coverage of the “French UMLS”; but the problem still remains.

Because the SIFR Annotator does not use machine translation, in order to obtain a fairer and more significant evaluation, we produced a pruned version of the Quaero gold-standard by filtering out all manual annotations made with CUIs for which there are no French labels in any of the 10 ontologies of the UMLS group in SIFR BioPortal. If all CUIs for a text span are removed, then the whole annotation is removed from the corpus. Table 2 presents the statistics of the original corpus compared to that of the adapted corpus. The script used to generate the subset of the corpus along with the list of CUIs used for the filtering will be made available on github.

**Table 2:** Number of CUIs expected between the gold standard annotations in the Quaero corpus and the adapted Quaero corpus

	Quaero		Adapted Quaero	
	EMEA Dev	MEDLINE Dev	EMEA Dev	MEDLINE Dev
CUIs (uniq.)	2261 (526)	2978 (1843)	1733 (425)	2465 (1477)
	EMEA Test	MEDLINE Test	EMEA Test	MEDLINE Test
CUIs (uniq.)	2203 (474)	3093 (1907)	1710 (388)	2606 (1544)
	EMEA Train	MEDLINE Train	EMEA Train	MEDLINE Train
CUIs (uniq.)	2695 (651)	2995 (1861)	2279 (541)	2491 (1488)

For the uniq. Statistic, only the first occurrence of a CUI is counted. In MEDLINE, each document is a title of 10–15 word forms on average, while EMEA documents are full notices with several hundred word forms each

#### Experimental Protocol & Parameters Tuning

We now present the experimental protocol used for the evaluation of SIFR Annotator on the EMEA and MEDLINE sub corpora of Quaero (original and adapted) on both the Plain Entity Recognition [PER] and the Normalized Entity Recognition [NER] annotations tasks, along with a description of the parameter tuning process. We present the baseline annotation setting along with two post-annotation disambiguation heuristics.

In the baseline setting, we used the “quaero” output format of the SIFR Annotator which produces a BRAT output format compliant with the evaluation scripts for the task. The parameters of SIFR Annotator used for the baseline annotation were the following:

- match_longest_only = false as the gold dataset annotates both multi-word terms and their constituents.
- match_patial_words = false as there are no such annotations possible in this task.
- negation = false, temporality = false, experiencer = false as the tasks does not require contextual annotations.
- semantic_groups = {}, semantic_types = {}, as all Semantic Types are found in the gold annotations.
- We used all the 10 terminologies in the UMLS group within the SIFR BioPortal.

Depending on the type of text we are annotating, using all 10 UMLS terminologies may not be ideal as some may not correspond to the data and thus create annotation noise (false positives). In the present evaluation, the EMEA and MEDLINE sub corpora contain very different types of text (citation titles vs. drug notices), which justifies the need of finding the best subset of ontologies. To that end, we performed a grid search over all combinations of terminologies (we evaluated a total of ${\sum}_{k=1}^{10}\left(\genfrac{}{}{0pt}{}{10}{k}\right)=1023$ combinations) by scoring the resulting annotations on each of the dev sub-corpora.^18^

Once the optimal combination is found for both MEDLINE and EMEA, we evaluated the performance of the baseline annotation and of two post-annotation disambiguation heuristics on the test and training corpora for both the original Quaero corpus and the adapted Quaero corpus. We report on the actual values of the optimal target ontology lists prior to the evaluation results in the next section.

Because the Quaero corpus was constructed considering the UMLS Metathesaurus as a unique semantic resource and given that the nature of the SIFR Annotator is to consider UMLS as a group of 10 terminologies, we can already predict a shortcoming of SIFR Annotator with regard to task performance. In UMLS, one concept from a particular source, may be tagged with more than one CUI and consequently to more than one Semantic Group, inevitably creating ambiguities when multiple sources are used together. This is a well-known constraint/limitation when using UMLS [23]. Most of the 10 UMLS source terminologies in SIFR BioPortal have concepts with multiple Semantic Groups and/or CUIs, whereas Quaero gold standard annotations used only one, which will predictably lead to an ambiguity problem. Additionally, given that an entity and its Semantic Group must be correct in PER before the CUI annotation in NER is counted as correct (as shown at the beginning of Section “Error Analysis”), we expect a decrease in precision, while recall should stay the same between PER and NER, similarly to all systems participating in CLEF eHealth 2015 Task 1 [Hypothesis 1].

Additionally, we can expect SIFR Annotator to perform better in terms of recall on the adapted Quaero corpus and thus a higher overall F1 score [Hypothesis 2].

#### Disambiguation heuristics

One way of mitigating the effect of the hypothesized compound effect of the ambiguity is to attempt to find a heuristic that avoids the ambiguity altogether at the potential expense of either precision or recall. We evaluated two heuristics:

- [DAA – Discard Ambiguous Annotations] If we favor precision over recall, then a strategy is to remove ambiguity altogether by discarding any annotations belonging to several Semantic Groups. This strategy will likely reduce recall as some of the discarded annotations could be true positives [Hypothesis 3].
- [DBP – Distribution Based Prioritization] If we favor recall over precision, then another strategy is to disambiguate the Semantic Groups by keeping the most likely group as estimated with regards to the development corpus. In other words, we learn a frequency-based ranking of Semantic Groups and always keep only the best ranking Semantic Group. Statistically, in many cases as far as word sense disambiguation is concerned, the most frequent sense of a word is correct a majority of times depending on the degree of ambiguity. The most frequent sense heuristic is typically used as a strong baseline in word sense disambiguation studies [67, 68]. Although in the case of Semantic Groups, the frequencies are not contextualized and thus will not impact as well as in a typical word sense disambiguation task, we expect some improvement in precision for PER and NER [Hypothesis 4].

#### Results

First, optimal parameters for both EMEA and MEDLINE are:

- Set of ontologies. This parameter is independent from the DAA and DBP heuristics.
- Ranking of Semantic Groups based on their frequency distribution in the development corpus (DBP heuristic).

Both parameters remain the same for the baseline on the full and adapted corpora.

Table 3 summarizes the optimal values of the parameters estimated on the Quaero development corpus.

**Table 3:** Estimated optimal parameters

	EMEA	MEDLINE
Optimal set of ontologies	MSHFRE, CIM-10, MDRFRE, SNMIFRE, CISP-2, CIF, ATCFRE	MSHFRE, MDRFRE, SNMIFRE, MEDLINE+, CIF, CISP-2, ATCFRE
Frequency ranking for Semantic Groups for DBP heuristic	CHEM, DISO, LIVB, PROC, ANAT, PHYS, OBJC, GEOG, DEVI, PHEN	DISO, PROC, ANAT, CHEM, LIVB, PHYS, DEVI, PHEN,GEOG, OBJC

We then ran the annotation for PER and NER, on EMEA and MEDLINE on the full and adapted Quaero training corpora with the baseline setting and with the two heuristics. Table 4 summarizes the results in terms of Precision (P), Recall (R), F1 measure, and provides the average and median values for other CLEF eHealth 2015 Task 1 participants to which we may compare our results.

**Table 4:** Results on the Quaero Training for PER and NER

	Plain Entity Recognition [PER]						Normalized Entity Recognition [NER]					
	P	R	F1	P	R	F1	P	R	F1	P	R	F1
	EMEA			EMEA adapted			EMEA			EMEA adapted		
BSL	64.0	51.7	57.2	63.1	59.3	61.2	49.8	30.9	37.8	48.6	35.1	40.8
DAA	58.3	51.6	54.8	57.5	59.3	58.4	45.0	30.7	36.2	44.0	34.8	38.8
DBP	**70.8**	**56.2**	**62.6**	**69.2**	**64.0**	**66.7**	**54.21**	**31.0**	**39.4**	**54.1**	**35.36**	**42.8**
Avg.	58.7	47.3	51.1	Not Available			33.3	46.0	34.7	Not Available		
Med.	73.1	55.9	61.3				19.1	56.5	25.2			
	MEDLINE			MEDLINE adapted			MEDLINE			MEDLINE adapted		
BSL	57.5	49.0	52.9	55.2	55.8	55.5	44.0	**30.5**	36.0	43.8	**35.5**	39.2
DAA	**67.9**	49.0	56.9	**62.2**	55.8	60.2	**52.9**	**30.5**	**38.7**	**52.7**	**35.5**	**42.4**
DBP	64.7	**54.0**	**58.9**	62.0	61.1	**61.5**	49.5	30.4	37.6	49.25	35.4	41.2
Avg.	53.3	39.6	44.0	Not Available			32.1	46.1	34.0	Not Available		
Med.	64.9	40.0	48.7				29.5	59.0	22.8			

Evaluation on both the EMEA and MEDLINE sub corpora for the original Quaero corpus and our adapted Quaero corpus. For the original corpora, we report on the average and median results of the systems participating in CLEF eHealth 2015 Task 1. Values in bold correspond to the best results in each category

##### PER evaluation

The baseline approach for PER obtains slightly better results (F1 = 57.2%) on the EMEA corpus compared to MEDLINE (F1 = 52,9%) which can probably be explained by the fact that each title in MEDLINE pertains to a broad range of medical topics whereas EMEA is only about medication notices. The former necessarily offers a more diverse distribution of Semantic Groups, which is more difficult to identify.

The DAA heuristic does not consistently lead to better results than the baseline. For EMEA, the performance is lower than the baseline (− 5.7%P, − 0.1%R, − 2.4%F1), while for MEDLINE, it significantly improves the baseline results (+ 9.1%P, + 0%R, + 4%F1). This seems to invalidate Hypothesis 3, as recall is unaffected. In EMEA, where there is less ambiguity, the heuristics tend to delete annotations where there was at least one correct Semantic Group annotation, which leads to lower precision, while for MEDLINE, it is more likely to delete annotations where none of the Semantic Groups are correct. With the DBP heuristic, there is a consistent increase in both P and R across EMEA (+ 6.8%P, + 4.5%R, + 5.4%F1) and MEDLINE (+ 7.2%P, + 5%R, + 6%F1), which validated Hypothesis 4, although there is also a reliable increase in recall.

Compared to CLEF eHealth 2015 Task 1 participants, on the EMEA sub-task, our system, in its best configuration, would rank 4th with regard to participating systems (− 4.3% compared to the system ranked right before and + 8.5% ahead of the following system) behind ERASMUS, IHS-RD and Watchdogs. Among those systems, the two with which we are methodologically comparable, ERASMUS and Watchdogs, both used some kind of machine translation approaches. IHS-RD (as well as the system right after ours) used supervised machine learning.

On the MEDLINE sub-task, SIFR Annotator would rank 2nd with regard to participating systems, only behind ERASMUS (+ 7.3%), but before IHS-RD (− 6.5%) and Watchdogs. This can be explained by the fact that MEDLINE has a set of more diverse expected Semantic Group annotations, while EMEA mostly contains CHEM and DISO, which means the entropy of the Semantic Group distribution is higher, which makes it more difficult to use a supervised machine learning. ERASMUS and SIFR Annotator being knowledge-based, they suffer much less from the increased entropy of the expected Semantic Group distribution. The advance of ERASMUS can mainly be explained by a richer dictionary enabled by the translation approach.

##### NER evaluation

As expected, due to the added difficulty of the NER task compared to PER, annotation performance is significantly lower. The drop (between − 16.9% F1 and 23.2% F1) is similar on average for all participating systems, which validates Hypothesis 1.

The relative effect of DAA and DBP is the same in PER and in NER, meaning that the ranking between the baseline and the two heuristics remains the same in NER than it was in PER.

For EMEA, the DBP heuristic performs best (39.4% F1), while for MEDLINE, DAA performs best (42.4% F1) due to a reduction in precision. This effect is understandable as the heuristics affect only the Semantic Group annotations and do not influence the FP and FN ratio in NER.

With regard to the ranking in CLEF eHealth 2015 Task 1, fewer systems participated. Without explanation, the IHS-RD system that outperformed us on EMEA in PER, completely fails to annotate with CUIs with a F1 score of less than 1%. We rank second after the ERASMUS system (− 25%) by far, however, SIFR Annotator is also much better than the other systems. Only HIT-W1 gets a F1 score above 1%, but SIFR Annotator is significantly ahead with + 17.6% (Supervised CRF with an UMLS sense inventory). The failure of supervised systems that did not use UMLS as a sense inventory is normal, given the small amount of training data compared to the millions of possible CUI annotation from UMLS and the label ambiguity. ERASMUS and SIFR Annotator do not suffer from this drawback. Despite the translation aspect, the better performance of ERASMUS is due to their superior coverage in PER but also because they annotate with UMLS CUIs directly as a target, while SIFR Annotator annotates with source concepts that are more ambiguous with regard to CUIs (we annotate many CUIs, while ERASMUS annotates only one as the task expects).

##### Evaluation with the adapted Quaero corpus

The overall effect of the adapted Quaero corpus on the results of the PER task is to slightly lower precision and significantly increased recall, which increases the F1 score, on average by + 3.9% on EMEA and by + 2.8% on MEDLINE. The overall effect on the NER task is similar but with a lower magnitude of change. The relative effects of the heuristics remain unchanged for both PER and NER. The adaptation of the corpus mostly has the expected effect of increasing the recall and thus the F1 score by a few points (Hypothesis 2). The decrease in precision indicates that on average the entities kept in the adapted corpus are more ambiguous in terms of CUIs compared to the full corpus. If we could evaluate all participating systems on the adapted corpus, we would expect that it does not affect the performance of translation-based systems, while there would be a consistent increase in the recall of systems that do not rely on translation approaches. This would likely bridge much of the gap with ERASMUS, while likely remaining second.

### Annotation of death certificates with ICD-10 codes

The objective of CLEF eHealth 2017 Task 2 [69] is to annotate death certificates with ICD-10 codes both in French and in American English. We chose to participate in the task in order to evaluate the performance of SIFR Annotator for French and the NCBO Annotator for English. Here, we only present the results for the French corpus and point to the system paper [50] for additional details. Let us first describe the task and the French corpus, followed by a presentation of the additional semantic sources used (SKOS dictionary) and of the algorithm that maps concept ICD-10 concept URLs to ICD codes.

#### Task and Corpus description

A corpus of French death certificates from CépiDC was provided: a training corpus of 65,844 documents and 195,204 lines,^19^ a development corpus of 27,851 document and 80,900 lines and a test corpus of 31,683 documents and 91,954 lines. The corpora are digitized versions of actual death certificates filled in by clinicians. Although the punctuation is not always correct or present, in the corpus, each document is already segmented in lines (as per the standard international death certificate model) which for the most part only contain single sentences.

The French corpus was provided in both an aligned and a raw format. We only report on the performance for the aligned corpus as our approach leads to similar results for both. The raw format provides two files, a CausesBrutes file and an Ident file. The former contains semicolon separated values for the Document identifier (DocID), the year the certificated was coded (YearCoded), the line identifier (LineID), the raw text as it appears in the certificate (RawText), an interval type during which the condition occurred (IntType - seconds, minutes, hours, weeks, years) and an interval value (IntValue). The Ident file contains a document identifier, the year the certificate was coded, the gender of the person, the code for the primary cause of death, the age and the location of death. Here is an example:

- DocID ; YearCoded; LineID; RawText; IntType; IntValue
- 161477; 2014 ; 1 ; INSUFFISANCE RESPIRATOIRE AIGUE; 3; 5
- 161477; 2014 ; 2 ; PNEUMOPATHIE D INHALATION; 3; 5
- 161477; 2014 ; 5 ; PSYCHOSE CHRONIQUE;NULL;NULL

- DocID; YearCoded; Gender; Age; LocationOfDeath
- 93715; 2014 ; 2 ; 80; 2

The performance on the task was reported as Precision, Recall and F1 score for the whole corpus and for the sub-corpus of deaths from external causes (a subset of ICD-10 codes), which are much harder to determine automatically. The baseline system produced by the organizers used conditional code frequencies estimated from the training data to select the most likely code for a death certificate line.

#### Dictionary construction

SIFR BioPortal already contained the French ICD-10^20^ (CIM-10) reference terminology. This OWL version was originally produced by the CISMeF team from an automatic export from the HeTOP server [32]. However, the purpose of ICD-10 is to serve as a general-purpose reference to code medical acts, and not to be directly used for text annotation and, especially not in a particular clinical task such as death certificate coding. Indeed, from our experiments, using the original CIM-10 alone for annotation leads to a F1 score below 10%.

For the French corpus, a set of dictionaries was provided by CépiDC that give a standardized description text of each of the codes that appear in the corpora. Additionally, the data from the aligned training and development corpora could also be used to enrich the lexical terms of ICD-10. In order to use these dictionaries within the SIFR Annotator, we had to encode them using a format accepted by SIFR BioPortal, which includes RDFS, OWL, SKOS, OBO or RRF (UMLS format). In this case, the ideal choice in terms of standardization, potential reusability and simplicity was to use SKOS (Simple Knowledge Organization System) a W3C Recommendation specialized for vocabularies and thesaurus. Thus, we produced a SKOS dictionary called CIM-10 DC based on the French dictionaries and aligned corpus.^21^

We set out in this construction process by first defining the appropriate schema to represent the SKOS dictionaries. We chose to use the same URIs as concepts identifiers for the skos:Concept than for the corresponding owl:Class in the available CIM-10 terminology, which allows our dictionaries to be fully aligned with the original terminologies they enrich (from the perspective of ontology alignment). Each of the CIM-10 codes was represented by a skos:Concept. The URIs are composed of a base URI and a class identifier that represents the CIM-10 codes, in the following format: “[A-Z][0–9][0–9]\.?[0–9]?” (e.g., G12.1 or A10).^22^

We first built a code index, that associated to each code to the list of labels retrieved from the DiagnosisText field in the dictionary; and then add text from the RawText and StandardText fields from the corpus (associated to codes through the ICD-10 field in the corpus file). For each code concept, the CépiDC dictionaries contained multiple labels. In order to follow SKOS specification, we had to select a preferred name automatically (skos:prefLabel) and assign the other labels as alternative labels (skos:altLabel), which has no consequence for annotation. The selection heuristic took the shortest label that does not contain three or more consecutive capital letter (likely an acronym).

An important issue when building the SKOS dictionaries was to assign ambiguous labels (i.e., identical labels which correspond to different codes). Indeed, those labels create ambiguity in the annotations and leads to better recall at the price of a low precision. For example, the label “choc septique” was present as preferred label or synonyms for 58 different codes. Our “ontological” approach posits that the same label should not be assigned to the same label, and yet ICD codes are not ontology concepts, but diagnostic codes, which shows a limit of semantic annotation approaches for such tasks, as opposed to machine learning systems that do not suffer from the same drawback.

We had to implement a selection heuristic to determine the most suitable code to which the label should be bound. Taking inspiration from the idea of the most frequent sense baseline often used in Word Sense Disambiguation tasks, we adopted a heuristic that assigns ambiguous labels to the most frequent code only (just like in the first evaluation). We use the training corpus to estimate the frequencies of use of the codes (gold standard annotations) so that when a label can belong to several codes, we can sort the codes by frequency and chose the most frequent code (MFC).

#### Mapping algorithm between concept URIs and ICD-10 codes

Given that we used the SIFR Annotator, besides manually curating the created SKOS dictionaries, the final step to obtaining a working system for the task was to write a complete workflow to^23^^:^

1. Read the corpus in the raw or aligned formats;
2. Send the text to the SIFR Annotator REST API with the right ontologies and annotation parameters and retrieve the annotations produced;
3. Apply post-annotation heuristics to reduce ambiguity;
4. Produce the output in the right raw or aligned format.

We have used only the “RawText” information of both the aligned and raw datasets. We did not use any other information/features such as age or gender contained in the files. The evaluation run performed the annotation with the longest_only parameter activated on a local instance of the SIFR Annotator with CIM-10 and the SKOS dictionary we produced as target ontologies. We implemented two post-annotation heuristics:

- *Most Frequent Code*. If a particular line was annotated with several codes, we only keep the most frequent code based on the code distribution of the training corpus.
- *Code Frequency Cutoff*. We calculate a normalized probability distribution of the codes that annotate a particular line and only keep the codes below a cumulative probability threshold.

However, both heuristics led to a stark reduction in recall without leading to a satisfactory increase in precision to compensate and thus ended up lowering the overall F1 scores, which is why we did not activate them for our participation in the task.

#### Results

13 runs have been submitted by 9 teams to the French raw evaluation. Seven runs have been submitted by five teams to the French aligned evaluation. Table 5 presents the results obtained by our SIFR Annotator against the average and median results of the runs submitted to the evaluation task.

**Table 5:** Results for ICD-10 coding of death certificates for the French Raw Evaluation

	All Causes			External Causes		
	P	R	F1	P	R	F1
SIFR	54.1	48.0	50.9	44.3	36.7	40.1
Avg.	47.5	35.8	40.6	36.7	24.7	29.2
Med.	54.1	41.4	50.8	44.3	28.3	37.6

We present P, R, F1 on all causes (all ICD-10 codes) and on external causes

The SIFR Annotator results are exactly the median value of all the results with the raw dataset, but slightly under the median value for the aligned datasets (all causes). Indeed, teams that have used other information from the aligned dataset probably performed better than the SIFR Annotator here. Regarding the external causes, we obtain better precision and F1 than the average and median results submitted to the challenge.

The other systems that participated on the French Raw task can be divided in three categories: supervised machine learning (TUC, LIMSI), information retrieval models (IMS-UNIPD, LITL) and annotation approaches (SIFR Annotator, SIBM, Mondeca). The official results and ranking only include SIBM, LITL, SIFR Annotator and TUC (with faulty submitted results). The unofficial systems include LIMSI, UNIPD, TUC (corrected) and Mondeca. The SIFR Annotator was ranked third on all causes and second on external causes behind the SIBM system. The SIBM system is significantly ahead (> 20 + %) as it is the only system to perform code disambiguation. The difference with the second system (LITL) and ours is only of + 0.1%, hardly a significant difference. Had LIMSI run officially with their supervised system, they would have been first (82.5% F1), followed by SIBM and then the corrected TUC system (between 66.6 and 66.7% F1) and UNIPD (between 44.1 and 53.7%).

The performance of SIFR Annotator is somewhat lower than for a typical entity recognition task, because of the significant ambiguity (the same label can correspond to several different classes (here ICD-10 codes) found in the dictionaries provided with the task and in turn in our SKOS dictionary. This highlights that such a focused and specific text mining task is most likely better suited for machine learning approaches. However, despite of their limitations, the NCBO and SIFR Annotators obtained median results, respectively on French and English, when compared to the performance of all the participating systems. Therefore, considering the other discussed advantages, we believe they are two services that can help in a wide class of text mining or annotation problems, but of course not for all.

### Clinical context detection evaluation

As described among the features of the SIFR Annotator, there is a module for contextualizing annotations (Negation, Experiencer, Temporality) based on the ConText algorithm [65]. We adapted the algorithm to French and enriched existing translation efforts. We evaluated the French ConText on a sub-corpus of death certificates from the CLEF eHealth Task 1 corpus (6 sentences for experiencer, 150 for temporality, 1030 for negation) and on a clinical corpus from the European Hospital Georges Pompidou (630 lines for experiencer, 475 lines for temporality, and 400 lines for negation). French ConText implementation & evaluation are described in another communication; hereafter, we briefly summarize the main results.^24^

We reported an evaluation of the SIFR Annotator with F1 scores between 83.7 & 86.3% for negated concepts (better by more than 5% of previously reported results adpating NegEx to French), F1 88.9% and 91.7% for the detection of historical entities and between 79.2 and 90.9% for concepts pertaining to an experiencer other than the patient. The results are on-par with other state-of-the-art approaches (NegEx for negation, machine learning, etc.), independently from the concept recognition performance. Please consult the full evaluation in the article for more details.

## Discussion

In this section we discuss the results of the three evaluations and explain some of the shortcomings of SIFR Annotator by reviewing typical errors made in the annotation process. Some of the limitations are task-specific, while others are more general. We shall then draw some perspectives for future improvements.

### Error analysis

In order to further improve our open web-service, we performed a detailed error analysis on the results of the two evaluation tasks from CLEF eHealth so as to be able to identify future direction for improvement. We reviewed and categorized the main errors in terms of False Positives and False negatives and give concrete examples from both tasks.

#### PER annotation errors

We extracted a list of 50 random errors from the outputs on the full Quaero corpus and looked at their causes in detail (Table 6).

**Table 6:** PER annotation error analysis

	Description	Example	% in EMEA (14 FP & 36 FN)	% in MEDLINE (15FP & 35 FN)
FP	Annotation with a concept that was not covered in the gold standard	“évaluant la douleur”/Proc. (i.e., “pain evaluation”) matched but not in gold standard.	**10**	10
	Partial annotation on some but not all of the expected tokens	“sytème nerveux central” recognized instead of “signes du système nerveux central” (spelling)	**10**	**12**
	Incorrect Semantic Group annotation	“rein” (kidney) annotated with DISO. instead of ANAT. Generates both an FP and an FN.	8	8
	Concept missing from the French ontologies in the portal	Expected annotation: “canaux” (canals), but the SIFR Annotator dictionary only contains “canal, sai” (canal unspecified), which cannot match	**34**	12
FN	Morphosyntactic variation	Expected annotation “sériques” (an adjectivation of sérum) as ANAT, whereas the ontology label is “sérum” (the noun).	18	**26**
	Formulation different from concept labels (synonym, paraphrase)	Expected annotation “flacon” (vial), while the ontology concept label read “bouteille” (bottle).	14	22
	Incorrect Semantic Group	“rein” (kidney) annotated with DISO instead of ANAT. Generates both an FP and an FN.	6	10
	Unrecognized acronym or medical abbreviation	The gold standard expects “SNM” to be annotated with DISO, while the ontologies only contain “syndrome malin des neuroleptiques”.	2	0

Performed on 50 uniformly sampled errors on EMEA and MEDLINE obtained with the baseline method. The two most common causes are highlighted in bold

Among the false positives, one of the most frequent cause of errors is the production of annotations that were not in the gold standard. Given that the creation of the gold standard is subjective in terms of the entities chosen to be annotated by the experts [15],^25^ such errors are caused because of the exhaustive automatic annotation performed, which is a positive characteristic for any annotation system. Without medical expertise, by looking at a subset of these annotations, we could obviously conclude that many of them were not actual errors but indeed missing annotations in the corpus. Such omissions constitute a bias playing against knowledge-based approaches, when the set of ontologies used to compile the dictionary is richer than what human annotators considered when building the gold standard. Conversely, machine learning approaches, trained directly on a subset of the annotated corpus will not encounter this problem, but on the other hand will not have the capability of generalizing on unseen text.

The other frequent false positive error, is when SIFR Annotator only annotates a concept partially i.e., annotates the individual words with separate concepts, but not the whole expected concept. The gold standard always annotates both multi-word terms and the individual constituents. The SIFR Annotator almost always get the individual words right but not the multi-word terms.

In the example given in Table 5, the label “signes du système nerveux central” (or a simplified/tokenized version of it) does not exist in the French UMLS terminologies. The corresponding preferred label of actual corresponding concept (matching Semantic Group and CUI) is: “signes et symptômes divers du système nerveux central” which means that human expertise was required to infer that the text corresponds to a broader concept, which is very hard to reproduce for the SIFR Annotator.

Such errors could be remedied by enriching the original terminologies and ontologies (or the compiled dictionary) with more alternative labels. As previously mentioned in Section “Terminology/Ontology Acquisition”, we are already working on this but have observed mitigated results where the gain in recall does not match the loose in precision for the moment.

The third most common cause of false positives is an incorrect Semantic Group annotation.^26^ For example, in some instances, we annotated with DISO (Disease), when it should be ANAT (Anatomy). Despite fixing some incoherent Semantic Type assignments in the source terminologies in the UMLS, the inevitable solution is to equip the SIFR Annotator with a multi-level (class, UMLS concept, type, group) disambiguation module. More generally, beyond ambiguity related to UMLS, the SIFR Annotator obviously suffers from ambiguity between the general usage of a word and its medical usage (e.g., cold).

Among false negatives, one of the most common causes of error is morphosyntactic variation (18%) or a different formulation of the labels compared to the text (14%), meaning variations of the word due to differing grammatical roles (plurals, conjugations, etc.) or a different formulation for complex concept labels. This limitation is inherent to the concept recognizer, Mgrep, that does not deal with such variations (see “canaux” example in Table 5). We are exploring two possible solutions to the problem:

- We have developed a beta lemmatization feature in the SIFR Annotator that is not yet properly evaluated. However preliminary tests indicate that it would fix morphosyntactic recognition errors significantly.
- We are developing an alternate concept recognizer robust to morphosyntactic variations and to reformulation of complex expression (based on stem indexing of the words of ontology labels and word-embedding matching), although the operational integration is not mature enough to permit a production-level evaluation like we have gone here.

A common error producing false negatives (34%) is the absence of a concept from the ontologies (with the adequate Semantic Type and CUI), which is mitigated to some extent with the adapted Quaero corpus as we remove CUIs that do not exist in French sources. In such cases, knowledge-based approaches are indeed intrinsically limited by their ability to recognize only entities that have been captured into knowledge inside ontologies or terminologies first.

Among the less-frequent causes of false negatives, we have ambiguous Semantic Group annotations that are the main cause of incorrect Semantic Groups in false positives already covered above. We thus come back to the same idea of a multi-level disambiguation approach as the best potential mitigation.

#### NER errors

Any of the PER errors above also lead to errors in the NER task as per the construction of the task itself along with additional errors caused by the finer grain annotation:

- (E1) The expected CUIs are present in the SIFR Annotator results, but there are additional CUI annotations, which generates TPs for the expected CUIs and FPs for the others.
- (E2) None of the CUI annotations match the expected CUIs, which leads to TNs being generated for the expected CUIs and FPs for the generated CUI annotations.

At least one CUI was found for all entities identified in PER. In EMEA, E1 corresponds to 40% errors and E2 corresponds to 60% of errors, while in MEDLINE, the proportion is 50/50. In the case of E1, a disambiguation of the multiple concepts returned by the SIFR Annotator would be an effective solution to the problem, as previously mentioned for ambiguous Semantic Groups annotations in PER. The main cause for E2 errors is that the expert annotators did not annotate with all possible CUIs but picked one CUI among many possibilities. Therefore, the SIFR Annotator might return more specific or more general concept, which are not incorrect but which result from different annotation perspectives.

#### Death certificate coding errors

Similarly, we sampled 200 false positives and false negatives from the best runs of the SIFR Annotator on the French aligned development dataset and proceeded to manually determine the causes of the errors (Table 7).

**Table 7:** Most frequent SIFR Annotator errors for the death certificate coding task at CLEF eHealth 2017

Error	Example	Percent
Formulation different from synonym labels for expected concept	“arrêt respiratoire” (R09.2) not identified in “arrêt cardio respiratoire” or “détresse cardiorespiratoire.”	79%
Morphosyntactic variation	“Arrêt respiratoire” (R09.2) not identified in text “arrët réspiratore” due to incorrect diacritic.	16%
Annotation with a more general code (higher in the concept hierarchy)	“coma d’origine indéterminée et arrêt respiratoire progressif” matched with a more specific code, while the gold standard expects “arrêt respiratoire” (R09.2)	2.5%
Correct annotation dependent of detecting implicit semantic information	Code I10 “hypertension essentielle (primitive)” is hard to identify from “TC suite à une chute avec épilepsie séquellaire et tr cognitifs” as expected in the gold standard. Code R68.8 “autres symptômes et signes généraux précisés” was not identified within the text “atteinte polyviscérale diffuse.”	2.5%

The most frequent types of error are the following (see examples in Table 7):

- (79%) Errors because of missing synonyms that cannot be matched at a string-match level.
- (16%) Morphosyntactic or lexical variation (e.g., accent, dash, comma, spelling). The errors due to morphosyntactic variation (and more general concept annotation due to a partial match) have the same cause that similar errors in the PER and NER evaluations and their possible solutions are the same: an alternative concept recognizer. The mapping expansion mechanism in SIFR Annotator could tackle such an issue, but there are very few mappings to and from CIM-10 at the moment. All phenomena that are common in reality but not captured as synonyms by the source ontologies will not be recognized properly.
- (2.5%) Annotations were made with a more specific code (i.e., child in ICD-10 hierarchy) compared to the gold standard, often because of a partial match within a phrase.
- (2.5%) Errors caused by implicit semantic information that requires medical knowledge to identify. In both examples in Table 7, the code to identify is very general and the text does not really convey the coding explicitly; perhaps other fields in the data or in the knowledge of the experts helped them to code this death certificate meaningfully. This issue can hardly be remedied in the context of the SIFR Annotator as it is a process at a higher order of complexity than merely performing concept annotations (complex semantic inference).

### Limitations & future prospects

The purpose of the SIFR Annotator, and originally of the NCBO Annotator [13, 24], was not to beat task-specific state-of-the-art annotation systems. The goal was to offer generic but quite accurate workflow directly connected to their respective ontology repository. The concrete advantages of the services come from: (i) the size and variety of their dictionaries coming from ontologies, (ii) their availability as a web service that can be easily included in any semantic indexing workflow, and finally (iii) their adoption of a semantic web vision that strongly encourages using dereferenceable URIs that can then be reused to facilitate data integration and semantic interoperability. One should also note that the semantic expansion step (which uses the mappings between ontologies and the is_a hierarchies to generate additional annotations) as well as the post-processing of the annotations (which scores and contextualizes the annotations) are interesting exclusive features that are evaluated neither with the Quaero corpus nor in CLEF eHealth 2017 task 1.

That being said, the main limitations we can draw from our evaluations and from the error analyses from the perspective of annotation tasks are the following:

- The concept recognition component (Mgrep) used in SIFR BioPortal is limited in some aspects compared to current state-of-the-art, however, it offers significant advantages in a few contexts. Mgrep favors precision over recall and has been shown to almost always outperform MetaMap [12]. Moreover, Mgrep is agnostic with regard to the annotating resources, while many other systems are coupled with the UMLS Metathesaurus only (e.g., MetaMap). Tools using more advanced NLP techniques (fuzzy matching, syntactic analysis, language model-based matching) can lead to equally precise annotations with an increased recall, but at the cost of execution speed. The main disadvantages of Mgrep are: simple string matching; closed-source and difficult to improve upon. Mgrep was chosen regardless of limitations because precision is more important than recall (for biomedical annotation) and in a production setting, the speed of the matching is of the utmost importance.^27^ Since we cannot contribute to Mgrep, the best course of action is the development of a new concept recognition component. Such a development is already underway and under active testing, for a potential release date in late 2018.
- The ontological resources publicly available for French are limited compared to resources for English and much work may be done to release new public ontologies and to engineer new ontologies for domains not covered by existing resources. Even since the inception of the SIFR project, this has been a major goal and an active effort, much more is needed. We are for instance collaborating with pharmacologists to build a comprehensive and legally recognized resource for medication and drugs in French that is interoperable with international ATC codes. We are also actively incorporating new terminologies and ontologies in the SIFR BioPortal. In the future we also plan to automatically enrich any semantic resources in the repository with Semantic Types using machine learning in order to continue to offer annotations at different level of granularity even for ontologies that have never been integrated in the UMLS.
- The SIFR BioPortal is a multi-ontology approach where all labels belong to a single dictionary, which leads to annotation ambiguities at different granularities (concepts, CUIs, Semantic Types or Groups). The SIFR Annotator therefore requires a multi-level disambiguation module, as previously discussed.

Besides those limitations, the SIFR Annotator has significant advantages that are not highlighted in the evaluation tasks. One advantage is the ability to exploit the hierarchy, to obtain an annotation of a text at different levels of semantic granularity, which in turn can be effectively exploited for indexing large amounts of biomedical or clinical data. Annotations of terms with higher level parents allows to capture a very broad thematic semantic information, and can be exploited for text classification, while more specific annotations can be used for general purpose indexing or for knowledge extraction.

Another advantage of SIFR BioPortal and Annotator is the ability for users to contribute mappings between ontologies. Mappings correspond to explicit equivalence relations between ontology concepts. The original BioPortal infrastructure supports the loading of explicit mappings between ontologies contained in the repository but also automatically generates mappings based on class labels, URIs or CUIs. Those mappings can be used for annotation. For example, to annotate with one target ontology (e.g., ICD-10 for diagnostic coding), while still benefiting from the labels and alternative labels accessible through mappings during the concept recognition phase.

SIFR BioPortal additionally supports interportal mappings that can refer to ontologies in NCBO-like ontology repository. In previous work, we have reconciled and uploaded in the SIFR BioPortal 228 K French/English interportal mappings for UMLS ontologies between SIFR and NCBO BioPortal [70]. In a multilingual context, in the future we could, for instance, annotate French text with English concepts (or vice versa) in order to generate comparable corpora indexes across languages (an invaluable resource for cross-lingual text mining and information retrieval).

Adapting the BioPortal technology to Spanish is a possible future extension of the SIFR Annotator technology. Not only does Spanish already have numerous medical ontologies and terminologies, but the potential impact for clinical text annotations that are interoperable between Spanish and English is extremely significant, especially in the context of the linguistic landscape in the United States, where Spanish speaking communities are an important demographic. As an example, such an adaptation would allow English-speaking doctors to access the essential information found in Spanish language clinical health records, when treating Spanish speaking patients. We are in the process of identifying relevant partners to concretize such project.

## Conclusions

We presented the development and evaluation of SIFR Annotator, a semantic free-text annotation service for French made available in the SIFR BioPortal ontology repository, based on technology from NCBO BioPortal. We adapted the technology for the French language and extended the original features to be more suitable for multi-level annotation of clinical text and other possible scenarios.

We have shown the SIFR Annotator web service is comparable, in terms of quality and annotation performance to other knowledge-based annotation approaches in the two presented tasks, although the task objectives were not directly compatible with our annotation approach.^28^ We believe that SIFR Annotator can help in a wide range of text mining or annotation problems, but of course not universally. We also highlighted the shortcomings of our SIFR Annotator tool and proposed some possible solutions for their mitigation in future technical evolutions of the service.

Our work on SIFR Annotator, is not limited to French, however, the technical efforts have mainly been focused on decoupling the architecture from English and for allowing an easy adaptation to other languages. Although our target language is French, we have made some of our new features also available for English [14] and we believe our efforts and experience would facilitate deployment of new instance of BioPortal and its Annotator in other language (especially roman language or linguistically close to French/English) after minor configuration and adjustments. Such an adaptation does not dispense from the gargantuan task of gathering and engineering ontologies in other languages, but it gives a platform to make the efforts meaningful.

SIFR BioPortal has become the largest generic and open –with publicly access resources, code and related data– French-language biomedical ontology and terminology repository in France. In turn, SIFR Annotator is today the richest French language open annotator web service (competing annotators are either not available or closed-source online services). We are currently developing several partnerships in France to use SIFR Annotator within hospitals (CHRU Nancy, George Pompidou European Hospital in Paris) or for large-scale annotation efforts (e.g., to annotate the corpus of course of the French national medicine curriculum in the SIDES 3.0 project).

## Availability and requirements

**Project name**: SIFR Annotator

**Web application**: http://bioportal.lirmm.fr/annotator

**Project home page**: http://www.lirmm.fr/sifr

**Code repository**: http://github.com/sifrproject

**NCBO codebase**: https://github.com/sifrproject/ncbo_annotator

Proxy: https://github.com/sifrproject/annotators

Operating system(s): The Web application is platform independent. An easy local deployment procedure is available using Docker to process sensitive (e.g., clinical) data in-house (https://github.com/sifrproject/docker-compose-bioportal). This works on Linux.

Programming language: Ruby 2.3 (NCBO codebase) + Java 8 (Proxy)

Other requirements: When deploying manually: Rails 4, Tomcat 8, Redis, Memcached, MySQL, Apache HTTP Sever + Phusion passenger. When deploying with Docker, a Linux system, Docker, Docker Compose.

License: Stanford NCBO code based is Licensed as BSD-2. LIRMM’s modification to codebase and Proxy’s implementation is open source (License not yet determined).

## Footnotes

1 Centre d’épidémiologie sur les causes médicales de décès, Unité Inserm US10, (http://www.cepidc.inserm.fr)

2 Article currently under review (second round) in Journal of Biomedical Informatics (JBI-17-745).

3 NCBO BioPortal hosts some non-English ontologies, most of the time (but not always) as “views” of their English counterparts.

4 The Resource Description Framework (RDF) is the W3C language to described data. It is the backbone of the semantic web. SPARQL is the corresponding query language. By adopting RDF as the underlying format, an ontology repository based on NCBO technology can easily make its data available as linked open data and queryable through a public SPARQL endpoint. To illustrate this, the reader may consult the Link Open Data cloud diagram (http://lod-cloud.net) that since 2017 includes ontologies imported from the NCBO BioPortal (most of the Life Sciences section).

5
www.bioontology.org/wiki/index.php/Category:NCBO_Virtual_Appliance

6 Not to be confused with the 28 terminologies in SIFR BioPortal.

7 Please note that NCBO Annotator is always present among the systems being compared in various reviews [58, 71, 72].

8 Named Entity Recognition (or entity extraction) is the process of locating and categorizing named entities in text. We consider semantic annotation, i.e., the process of locating concepts previously defined in ontologies into text, as a subtask of NER.

9 For WHOFRE and ICPCFRE we had another version and LNC-FR-FR has not been included yet because it has a specific format.

10 CISMeF’s repository is larger, but the content is not publicly accessible.

11
https://goo.gl/rccsJi

12
https://goo.gl/5mJmgv

13 You may refer to the API documentation: http://data.bioportal.lirmm.fr/documentation.

14 The filtering by Semantic Type was available in the core Annotator components, however, we extended this feature to Semantic Groups which are themselves defined by grouping Semantic Types [14].

15 Since BioPortal 4.0 (end of 2013), the scoring has been removed from the NCBO Annotator. In our work we have re-implemented the original score and offered two better ones.

16 For instance, with an absolute score threshold of 3.1: https://goo.gl/yKe8gY

17 Proxy in the sense of the architectural software design principle applied to microservice architectures, not to be confused with an HTTP Proxy, a tool to secure external internet access from within a closed network.

18 All parameter estimation efforts are performed on the original Quaero corpus with the aforementioned baseline parameters.

19 Each death certificate is a document and contains exactly 4 lines.

20
http://bioportal.lirmm.fr/ontologies/CIM-10

21 We are currently interacting with the CéPIDC to potentially publicly release the dictionary we’ve build for third parties.

22 The corresponding URI in CIM-10 and thus in CIM-10 DC is http://chu-rouen.fr/cismef/CIM-10#G12.1, where http://chu-rouen.fr/cismef/CIM-10# is the base URI and G12.1 the code identifier.

23 The evaluation program for the death certificate coding task was implemented in Java, in the same repository as for the named entity recognition evaluation on the Quaero corpus.

24 A first version of the system has been published in a French peer-reviewed workshop [17]. A more complete evaluation and system description is currently under review (second round) for JBI.

25 Annotators were supplied with entity-pre-annotations but were given free rein to delete annotations or add new entity annotations. The paper reports that a significant number of entities were added after the pre-annotation process, however, exhaustivity is difficult to achieve.

26 The 10% breakdown into 2% incorrect single semantic group annotations and 8% ambiguous annotations with multiple semantic groups.

27 An experimental recognizer based on Mallet and AlvisNLP have been built in the context of a past hackathon but proved incompatible with a production-grade annotation system due to their slow processing speed.

28 It is important to note our system was not specifically tailored for these tasks and that performance will highly vary depending of the data to annotate and the ontologies targeted.
